# (3*Z*)-4-Methyl-9-(4-methyl­benzene­sulfon­yl)-*N*-phenyl-3*H*,9*H*-thio­pyrano[3,4-*b*]indol-3-imine

**DOI:** 10.1107/S2414314625004754

**Published:** 2025-05-30

**Authors:** Benjamin Dassonneville, Heiner Detert, Dieter Schollmeyer

**Affiliations:** a University of Mainz, Department of Chemistry, Duesbergweg 10-14, 55099 Mainz, Germany; Goethe-Universität Frankfurt, Germany

**Keywords:** crystal structure, heterocarbazole, thio­pyrane

## Abstract

The title indolothiopyrane imine, C_25_H_20_N_2_O_2_S_2_, which was prepared by a [2 + 2 + 2] cycloaddition reaction, crystallizes with two molecules in the asymmetric unit. Both adopt the shape of a staircase with two steps, consolidated by intramolecular C—H⋯O interactions.

## Graphical abstract

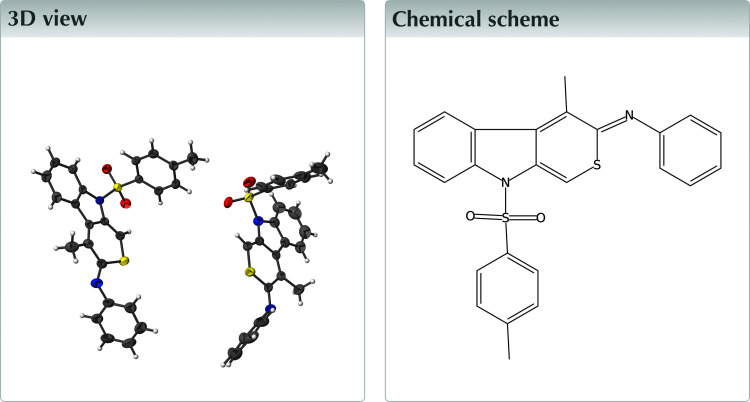

## Structure description

**Figure 1:**
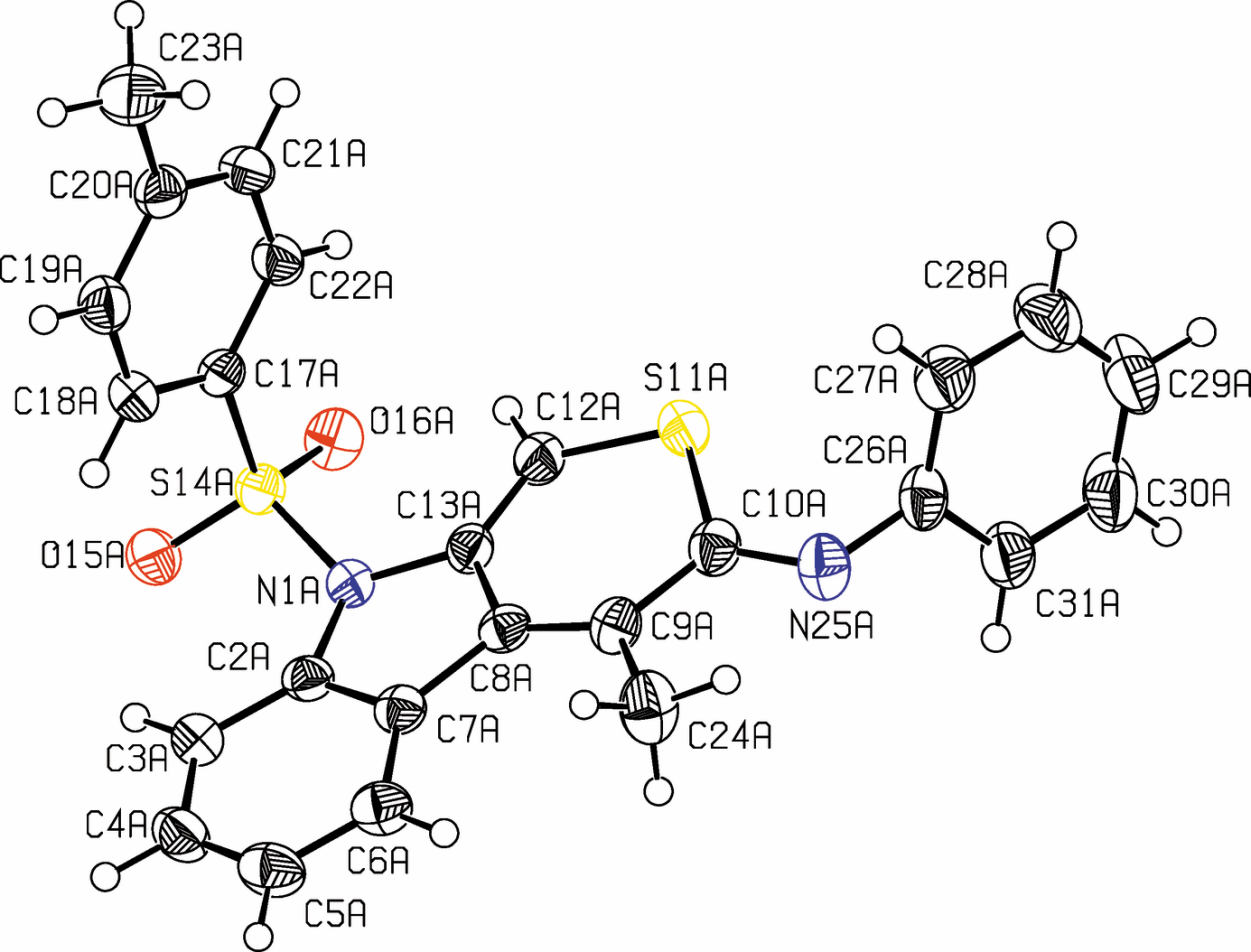
View of the title compound. Displacement ellipsoids are drawn at the 50% probability level. Only one of the two independent mol­ecules is shown.

**Figure 2:**
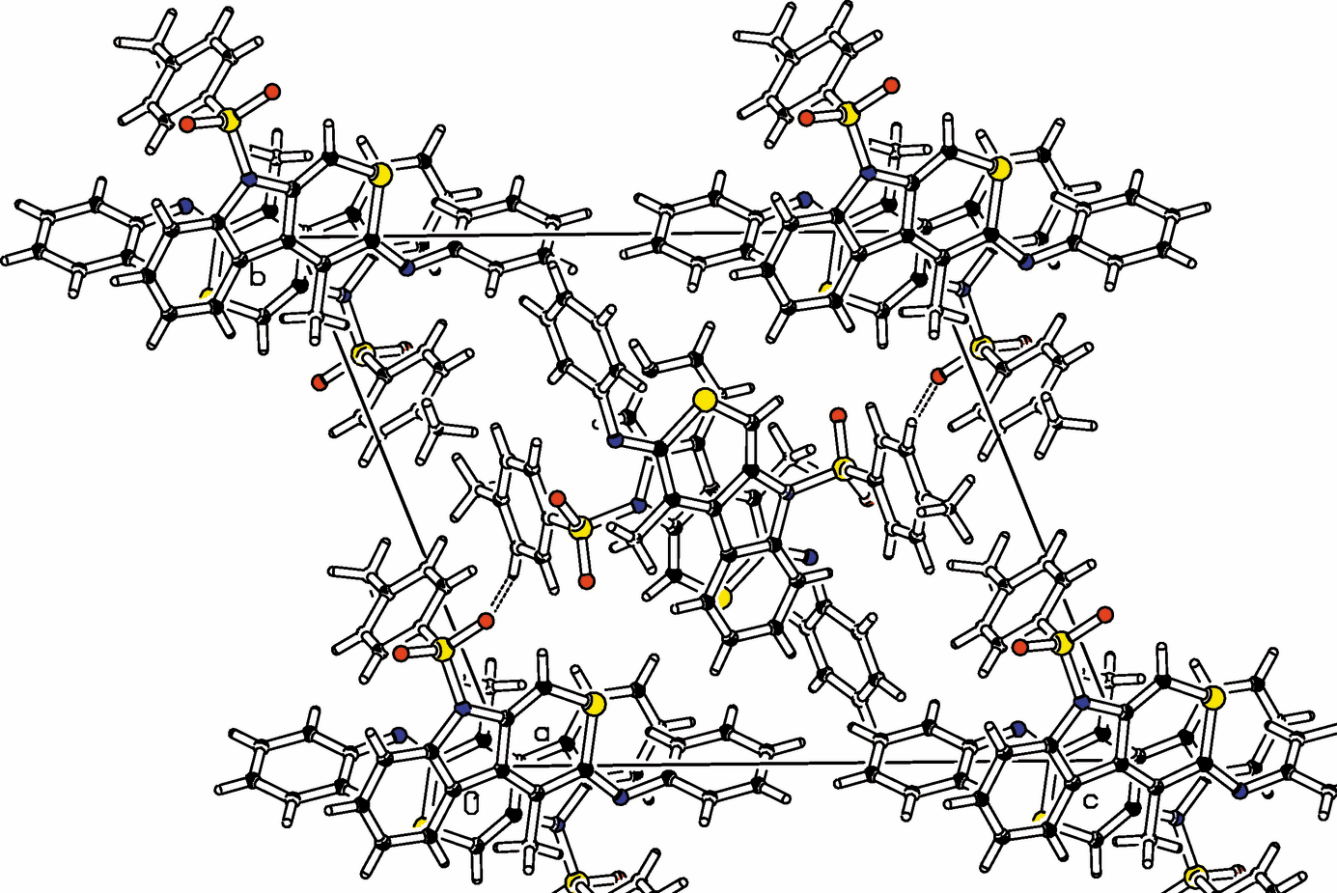
Partial packing diagram viewed along the *a*-axis direction. The contact O16*B*⋯H21*A* is shown as a dashed line.

The title compound (Fig. 1 ▸) was prepared in a larger project focusing on heterocarbazoles (Dassonneville *et al.*, 2011 ▸; Letessier *et al.*, 2012 ▸; 2013 ▸; Letessier & Detert, 2012 ▸). Similar to the preparation of indolo­thio­pyran­ethio­nes (Dassonneville *et al.* 2023*a* ▸,*b* ▸), a rhodium-catalyzed 2 + 2 + 2 cyclo­addition of an *O*,*N*-dialkynyl-*N*-tosyl­aniline and phenyl­iso­thio­cyanate formed the heterocycle of the indolo­thio­pyrane imine, C_25_H_20_N_2_O_2_S_2_, in 31% yield. The indolo­thio­pyran segment is close to planar, with a maximum deviation from the mean plane of 0.1267 (16) Å for C10*A* and 0.1361 (17) Å for C10*B*. An angle of 81.12 (6)° is enclosed by the planes of the thia­carbazole and the phenyl ring in mol­ecule *A* and 74.88 (5)° in mol­ecule *B*. The tolyl ring and the thia­carbazole moiety enclose an angle of 87.42 (5)° in mol­ecule *A* and 83.35 (5)° in mol­ecule *B*. The cyclic substituents are on opposite sides of the tricyclic ring system; they enclose an angle of 16.33 (14)° in mol­ecule *A* and 37.48 (8)° in mol­ecule *B*. The crystal packing of the mol­ecules is consolidated by weak C—H⋯O inter­actions (Fig. 2 ▸, Table 1 ▸). Two very similar mol­ecules (*A*,*B*) fill the unit cell. *A* and *B* are connected by an inter­molecular H-bridge from C21*A* to O16*B*, the distance H21*A*⋯O16*B* is 2.45 Å. The crystal is composed of centrosymmetrical pairs *A*,*A* and *B*,*B*. Within the *A*,*A* pair, one *o*-hydrogen atom (H27*A*) of the phenyl ring points to the center of the benzo unit (C2*A*–C7*A*) of the heterocarbazole. The H27*A*⋯centroid distance is 2.89 Å. The *B*–*B* pair has a H27*B*⋯centroid distance of 2.95 Å.

## Synthesis and crystallization

2-Propin-1-yl-*N*-tosyl-*N*-ethynylaniline was prepared according to the literature (Dassonneville *et al.*, 2023*a* ▸).

*Activation of catalyst*: a dry Schlenk tube was filled with nitro­gen, 2.5 mol-% (4.6 mg) [RhCl(COD)]_2_, 6 mol-% (9.6 mg) BINAP and 3 ml CH_2_Cl_2_ and hydrogen was bubbled through the solution. After the color changed, the solvent was evaporated *in vacuo*.

*[2 + 2 + 2] Cyclo­addition:* the catalyst was dissolved in 5 ml of di­chloro­ethane, a solution of the diyne (78.8 mg, 0.255 mmol) and phenyl iso­thio­cyanate (230 mg, 2.55 mmol) in 7 ml of dichloethane (DCE) was added *via* syringe. After 24 h at 80°C, the solvent was removed and the residue purified *via* chromatography [SiO_2_, (1) petroleum ether: ethyl acetate 1:1 (*v*:*v*), (2) ethanol]. Separation of product and carbamate was *via* crystallization of the latter from DCE/petroleum ether solution. *R*_f_: 0.46 (SiO_2_, petroleum ether/ethyl acetate = 9/1) gave 35 mg (31%) *N*-(1-methyl-9-tosyl­thio­pyrano[3,4-*b*]indol-3(9*H*)-yliden)aniline as a red–orange solid with m.p.= 456–457 K. The annotation of the NMR signals follows IUPAC nomenclature. ^1^H-NMR (400 MHz, CDCl_3_): δ p.p.m.: 8.15 (*d*, *J* = 8.2 Hz, 1H), 7.95 (*d*, *J* = 7.9 Hz, 1H), 7.60 (*br. s*, 1H), 7.55 (*d*, *J* = 8.4 Hz, 2H), 7.51 (*t*, *J* = 7.4 Hz, 1H), 7.43 (*t*, *J* = 7.6 Hz, 2H), 7.30 (*t*, *J* = 8.1 Hz, 1H), 7.16 (*d*, *J* = 8.2 Hz, 2H), 7.14 (*t*, *J* = 9.7 Hz, 1H), 6.90 (*d*, *J* = 7.7 Hz, 2H), 2.36 (*s*, 3H), 2.34 (*s*, 3H); ^13^C-NMR (100 MHz, CDCl_3_): 145.5 (C_q_), 142.0 (C_q_), 133.5 (C_q_), 131.2 (CH), 130.7 (C_q_), 130.3 (CH’), 129.9 (CH), 128.7 (C_q_), 126.9 (CH), 126.8 (C_q_), 126.2 (CH), 125.1 (CH), 124.8 (CH), 120.1 (CH), 115.7 (CH), 109.0 (CH), 21.6 (CH_3_), 15.8 (CH_3_). C_q_ missing due to overlapping. IR (neat, ATR) = 2922, 2850, 1733, 1532, 1456, 1372, 1264, 1095, 800 cm^−1^; FD–MS: *m*/*z* (%) = 444.3 (100) [*M*^+^], 445.3 (34), 446.3 (17). Exact mass: [*M* + H]^+^ calculated: 445.1044; found 445.1039.

## Refinement

Crystal data, data collection and structure refinement details are summarized in Table 2 ▸.

## Supplementary Material

Crystal structure: contains datablock(s) I, global. DOI: 10.1107/S2414314625004754/bt4173sup1.cif

Structure factors: contains datablock(s) I. DOI: 10.1107/S2414314625004754/bt4173Isup2.hkl

Supporting information file. DOI: 10.1107/S2414314625004754/bt4173Isup3.cml

CCDC reference: 2453955

Additional supporting information:  crystallographic information; 3D view; checkCIF report

Additional supporting information:  crystallographic information; 3D view; checkCIF report

## Figures and Tables

**Table 1:** Hydrogen-bond geometry (Å, °)

*D*—H⋯*A*	*D*—H	H⋯*A*	*D*⋯*A*	*D*—H⋯*A*
C3*A*—H3*A*⋯O15*A*	0.95	2.25	2.878 (3)	123
C3*B*—H3*B*⋯O15*B*	0.95	2.25	2.871 (3)	122
C12*A*—H12*A*⋯O16*A*	0.95	2.23	2.909 (2)	127
C12*B*—H12*B*⋯O16*B*	0.95	2.24	2.922 (3)	128
C21*A*—H21*A*⋯O16*B*	0.95	2.45	3.366 (3)	161
C22*A*—H22*A*⋯O16*A*	0.95	2.55	2.923 (2)	104
C22*B*—H22*B*⋯O16*B*	0.95	2.51	2.900 (3)	104
C24*A*—H24*B*⋯N25*A*	0.98	2.31	2.754 (4)	106
C24*B*—H24*E*⋯N25*B*	0.98	2.27	2.744 (3)	108

**Table 2:** Experimental details

Crystal data	
Chemical formula	C_25_H_20_N_2_O_2_S_2_
*M*_r_	444.55
Crystal system, space group	Triclinic, *P* 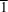
Temperature (K)	173
*a*, *b*, *c* (Å)	9.1997 (6), 15.1692 (8), 16.7138 (9)
α, β, γ (°)	110.1289 (13), 100.7495 (16), 94.2372 (15)
*V* (Å^3^)	2127.3 (2)
*Z*	4
Radiation type	Mo *K*α
μ (mm^−1^)	0.28
Crystal size (mm)	0.40 × 0.18 × 0.09

Data collection	
Diffractometer	Bruker SMART APEXII
No. of measured, independent and observed [*I* > 2σ(*I*)] reflections	29569, 10121, 7293
*R*_int_	0.035
(sin θ/λ)_max_ (Å^−1^)	0.658

Refinement	
*R*[*F*^2^ > 2σ(*F*^2^)], *wR*(*F*^2^), *S*	0.044, 0.120, 1.01
No. of reflections	10121
No. of parameters	563
H-atom treatment	H-atom parameters constrained
Δρ_max_, Δρ_min_ (e Å^−3^)	0.78, −0.41

Computer programs: *SMART* and *SAINT* (Bruker (1997 ▸), *SIR2004* (Altomare *et al.*, 1999 ▸), *SHELXL2019/2* (Sheldrick, 2015 ▸) and *PLATON* (Spek, 2009 ▸).

## full crystallographic data

### (3Z)-4-Methyl-9-(4-methylbenzenesulfonyl)-N-phenyl-3H,9H-thiopyrano[3,4-b]indol-3-imine Crystal data

**Table:**

C_25_H_20_N_2_O_2_S_2_	*Z* = 4
*M**_r_* = 444.55	*F*(000) = 928
Triclinic, *P*1	*D*_x_ = 1.388 Mg m^−^^3^
*a* = 9.1997 (6) Å	Mo *K*α radiation, λ = 0.71073 Å
*b* = 15.1692 (8) Å	Cell parameters from 6518 reflections
*c* = 16.7138 (9) Å	θ = 2.2–27.5°
α = 110.1289 (13)°	µ = 0.28 mm^−^^1^
β = 100.7495 (16)°	*T* = 173 K
γ = 94.2372 (15)°	Needle, orange
*V* = 2127.3 (2) Å^3^	0.40 × 0.18 × 0.09 mm

### (3Z)-4-Methyl-9-(4-methylbenzenesulfonyl)-N-phenyl-3H,9H-thiopyrano[3,4-b]indol-3-imine Data collection

**Table:**

Bruker SMART APEXII diffractometer	7293 reflections with *I* > 2σ(*I*)
Radiation source: sealed tube	*R*_int_ = 0.035
Graphite monochromator	θ_max_ = 27.9°, θ_min_ = 1.6°
CCD scan	*h* = −12→12
29569 measured reflections	*k* = −19→19
10121 independent reflections	*l* = −20→22

### (3Z)-4-Methyl-9-(4-methylbenzenesulfonyl)-N-phenyl-3H,9H-thiopyrano[3,4-b]indol-3-imine Refinement

**Table:**

Refinement on *F*^2^	Primary atom site location: structure-invariant direct methods
Least-squares matrix: full	Hydrogen site location: inferred from neighbouring sites
*R*[*F*^2^ > 2σ(*F*^2^)] = 0.044	H-atom parameters constrained
*wR*(*F*^2^) = 0.120	*w* = 1/[σ^2^(*F*_o_^2^) + (0.0548*P*)^2^ + 0.7083*P*] where *P* = (*F*_o_^2^ + 2*F*_c_^2^)/3
*S* = 1.01	(Δ/σ)_max_ = 0.001
10121 reflections	Δρ_max_ = 0.78 e Å^−^^3^
563 parameters	Δρ_min_ = −0.41 e Å^−^^3^
0 restraints	

### (3Z)-4-Methyl-9-(4-methylbenzenesulfonyl)-N-phenyl-3H,9H-thiopyrano[3,4-b]indol-3-imine Special details

**Table:**

Geometry. All esds (except the esd in the dihedral angle between two l.s. planes) are estimated using the full covariance matrix. The cell esds are taken into account individually in the estimation of esds in distances, angles and torsion angles; correlations between esds in cell parameters are only used when they are defined by crystal symmetry. An approximate (isotropic) treatment of cell esds is used for estimating esds involving l.s. planes.
Refinement. Hydrogen atoms were placed at calculated positions and were refined in the riding-model approximation with C_aromatic_–H = 0.95 Å and with *U*_iso_(H) = 1.2 *U*_eq_(C) or C_methyl_–H = 0.98 Å, and with *U*_iso_(H) = 1.5 *U*_eq_(C). The methyl groups were allowed to rotate but not to tip.

### (3Z)-4-Methyl-9-(4-methylbenzenesulfonyl)-N-phenyl-3H,9H-thiopyrano[3,4-b]indol-3-imine Fractional atomic coordinates and isotropic or equivalent isotropic displacement parameters (Å^2^)

**Table:**

	*x*	*y*	*z*	*U*_iso_*/*U*_eq_	
N1A	0.04721 (19)	0.48944 (11)	0.37672 (10)	0.0314 (4)	
C2A	0.0590 (2)	0.58682 (13)	0.43169 (12)	0.0308 (4)	
C3A	−0.0219 (2)	0.65539 (15)	0.41690 (14)	0.0382 (5)	
H3A	−0.091966	0.642317	0.363233	0.046*	
C4A	0.0034 (3)	0.74376 (15)	0.48341 (15)	0.0433 (5)	
H4A	−0.048670	0.792537	0.474345	0.052*	
C5A	0.1021 (3)	0.76293 (15)	0.56246 (15)	0.0452 (5)	
H5A	0.114933	0.823746	0.607353	0.054*	
C6A	0.1824 (2)	0.69408 (15)	0.57670 (14)	0.0392 (5)	
H6A	0.249815	0.707268	0.631311	0.047*	
C7A	0.1637 (2)	0.60505 (14)	0.51021 (12)	0.0312 (4)	
C8A	0.2275 (2)	0.51789 (13)	0.50494 (12)	0.0304 (4)	
C9A	0.3307 (2)	0.50056 (14)	0.56565 (13)	0.0347 (4)	
C10A	0.3678 (2)	0.40504 (15)	0.55271 (13)	0.0367 (5)	
S11A	0.31344 (7)	0.31426 (4)	0.44779 (4)	0.04232 (14)	
C12A	0.1845 (2)	0.35787 (15)	0.38829 (13)	0.0364 (5)	
H12A	0.132372	0.317313	0.331114	0.044*	
C13A	0.1549 (2)	0.44693 (14)	0.41971 (12)	0.0303 (4)	
S14A	−0.01665 (5)	0.44661 (3)	0.26855 (3)	0.03142 (12)	
O15A	−0.12706 (16)	0.50250 (11)	0.25037 (9)	0.0398 (3)	
O16A	−0.05747 (17)	0.34670 (10)	0.24397 (9)	0.0405 (3)	
C17A	0.1364 (2)	0.46768 (13)	0.22566 (11)	0.0292 (4)	
C18A	0.1790 (2)	0.55859 (14)	0.22872 (13)	0.0347 (4)	
H18A	0.125516	0.608657	0.253205	0.042*	
C19A	0.3004 (2)	0.57493 (15)	0.19551 (14)	0.0387 (5)	
H19A	0.330427	0.636964	0.197226	0.046*	
C20A	0.3799 (2)	0.50230 (16)	0.15956 (13)	0.0370 (5)	
C21A	0.3331 (2)	0.41169 (15)	0.15598 (13)	0.0381 (5)	
H21A	0.385417	0.361341	0.130556	0.046*	
C22A	0.2120 (2)	0.39345 (14)	0.18873 (12)	0.0352 (4)	
H22A	0.180881	0.331184	0.186067	0.042*	
C23A	0.5161 (3)	0.5223 (2)	0.12750 (17)	0.0544 (6)	
H23A	0.606216	0.529419	0.172362	0.082*	
H23B	0.513026	0.581057	0.115455	0.082*	
H23C	0.517940	0.469536	0.073783	0.082*	
C24A	0.4067 (3)	0.57533 (17)	0.65269 (15)	0.0509 (6)	
H24A	0.445393	0.632492	0.643979	0.076*	
H24B	0.489644	0.551405	0.680865	0.076*	
H24C	0.334568	0.590850	0.689978	0.076*	
N25A	0.4383 (2)	0.38758 (14)	0.61811 (12)	0.0451 (5)	
C26A	0.4692 (2)	0.29518 (16)	0.60954 (14)	0.0418 (5)	
C27A	0.5790 (3)	0.25582 (19)	0.56890 (17)	0.0527 (6)	
H27A	0.631147	0.289099	0.541413	0.063*	
C28A	0.6136 (3)	0.1683 (2)	0.5678 (2)	0.0632 (7)	
H28A	0.689697	0.142053	0.539834	0.076*	
C29A	0.5390 (3)	0.1192 (2)	0.6069 (2)	0.0626 (7)	
H29A	0.562695	0.058947	0.605950	0.075*	
C30A	0.4297 (3)	0.1580 (2)	0.64749 (19)	0.0630 (7)	
H30A	0.376979	0.124022	0.674158	0.076*	
C31A	0.3954 (3)	0.24613 (19)	0.65005 (17)	0.0527 (6)	
H31A	0.321206	0.272907	0.679563	0.063*	
N1B	0.5849 (2)	0.11035 (12)	−0.03785 (12)	0.0389 (4)	
C2B	0.5523 (2)	0.02961 (15)	−0.11763 (14)	0.0402 (5)	
C3B	0.4545 (3)	0.01695 (18)	−0.19568 (17)	0.0539 (6)	
H3B	0.399483	0.065558	−0.202339	0.065*	
C4B	0.4397 (3)	−0.0698 (2)	−0.26406 (18)	0.0616 (7)	
H4B	0.375848	−0.079614	−0.319031	0.074*	
C5B	0.5149 (3)	−0.14121 (19)	−0.25407 (17)	0.0594 (7)	
H5B	0.502002	−0.199723	−0.301958	0.071*	
C6B	0.6093 (3)	−0.12937 (16)	−0.17531 (16)	0.0487 (6)	
H6B	0.658470	−0.180031	−0.168331	0.058*	
C7B	0.6317 (2)	−0.04174 (14)	−0.10569 (14)	0.0380 (5)	
C8B	0.7225 (2)	−0.00659 (13)	−0.01636 (13)	0.0343 (4)	
C9B	0.8153 (3)	−0.05090 (14)	0.02560 (14)	0.0390 (5)	
C10B	0.8845 (3)	−0.00796 (14)	0.11901 (14)	0.0374 (5)	
S11B	0.88750 (7)	0.11530 (4)	0.17585 (4)	0.04207 (14)	
C12B	0.7609 (3)	0.14905 (14)	0.10594 (14)	0.0378 (5)	
H12B	0.737776	0.211879	0.125847	0.045*	
C13B	0.6941 (2)	0.09021 (14)	0.02483 (13)	0.0342 (4)	
S14B	0.57056 (6)	0.22115 (4)	−0.03227 (4)	0.03805 (13)	
O15B	0.44857 (18)	0.21419 (12)	−0.10166 (12)	0.0539 (4)	
O16B	0.56583 (18)	0.27399 (11)	0.05614 (11)	0.0484 (4)	
C17B	0.7374 (2)	0.26277 (14)	−0.05261 (13)	0.0333 (4)	
C18B	0.7710 (3)	0.21880 (15)	−0.13335 (16)	0.0455 (6)	
H18B	0.704340	0.166844	−0.177597	0.055*	
C19B	0.9034 (3)	0.25224 (17)	−0.14790 (18)	0.0539 (7)	
H19B	0.927828	0.222265	−0.202737	0.065*	
C20B	1.0015 (3)	0.32839 (19)	−0.08460 (19)	0.0526 (6)	
C21B	0.9635 (3)	0.3720 (2)	−0.00578 (17)	0.0547 (7)	
H21B	1.028335	0.425561	0.037600	0.066*	
C22B	0.8327 (3)	0.33931 (17)	0.01124 (15)	0.0443 (5)	
H22B	0.808702	0.369169	0.066237	0.053*	
C23B	1.1475 (3)	0.3622 (3)	−0.1015 (2)	0.0796 (10)	
H23D	1.128407	0.370361	−0.157994	0.119*	
H23E	1.215342	0.315164	−0.102407	0.119*	
H23F	1.193505	0.423016	−0.054939	0.119*	
C24B	0.8473 (3)	−0.15006 (16)	−0.01953 (16)	0.0553 (7)	
H24D	0.755078	−0.195395	−0.037102	0.083*	
H24E	0.922891	−0.166211	0.020704	0.083*	
H24F	0.884531	−0.152861	−0.071463	0.083*	
N25B	0.9425 (2)	−0.06027 (13)	0.15837 (12)	0.0445 (5)	
C26B	1.0101 (3)	−0.02585 (15)	0.24820 (15)	0.0414 (5)	
C27B	1.1430 (3)	0.03578 (17)	0.28392 (17)	0.0524 (6)	
H27B	1.187374	0.062140	0.248629	0.063*	
C28B	1.2118 (3)	0.05929 (18)	0.37085 (19)	0.0610 (7)	
H28B	1.303256	0.101967	0.394939	0.073*	
C29B	1.1502 (4)	0.0220 (2)	0.42261 (18)	0.0634 (8)	
H29B	1.197866	0.038921	0.482484	0.076*	
C30B	1.0187 (4)	−0.0402 (2)	0.38730 (19)	0.0676 (8)	
H30B	0.975712	−0.066580	0.423004	0.081*	
C31B	0.9483 (3)	−0.06469 (19)	0.30064 (16)	0.0535 (6)	
H31B	0.857618	−0.108095	0.276788	0.064*	

### (3Z)-4-Methyl-9-(4-methylbenzenesulfonyl)-N-phenyl-3H,9H-thiopyrano[3,4-b]indol-3-imine Atomic displacement parameters (Å^2^)

**Table:**

	*U*^11^	*U*^22^	*U*^33^	*U*^12^	*U*^13^	*U*^23^
N1A	0.0364 (9)	0.0305 (8)	0.0289 (8)	0.0082 (7)	0.0088 (7)	0.0115 (7)
C2A	0.0340 (10)	0.0289 (10)	0.0322 (10)	0.0050 (8)	0.0139 (8)	0.0109 (8)
C3A	0.0434 (12)	0.0376 (11)	0.0375 (11)	0.0126 (9)	0.0125 (9)	0.0156 (9)
C4A	0.0491 (13)	0.0335 (11)	0.0533 (14)	0.0142 (10)	0.0193 (11)	0.0175 (10)
C5A	0.0512 (14)	0.0296 (11)	0.0494 (13)	0.0043 (10)	0.0166 (11)	0.0054 (10)
C6A	0.0412 (12)	0.0341 (11)	0.0378 (11)	0.0008 (9)	0.0102 (9)	0.0078 (9)
C7A	0.0311 (10)	0.0309 (10)	0.0340 (10)	0.0021 (8)	0.0118 (8)	0.0131 (8)
C8A	0.0314 (10)	0.0307 (10)	0.0307 (10)	0.0005 (8)	0.0110 (8)	0.0119 (8)
C9A	0.0352 (11)	0.0346 (11)	0.0335 (10)	−0.0003 (9)	0.0048 (8)	0.0143 (9)
C10A	0.0329 (11)	0.0420 (12)	0.0376 (11)	0.0045 (9)	0.0065 (9)	0.0186 (9)
S11A	0.0517 (3)	0.0384 (3)	0.0396 (3)	0.0171 (2)	0.0095 (2)	0.0159 (2)
C12A	0.0448 (12)	0.0348 (11)	0.0300 (10)	0.0079 (9)	0.0081 (9)	0.0120 (9)
C13A	0.0314 (10)	0.0329 (10)	0.0298 (9)	0.0049 (8)	0.0082 (8)	0.0148 (8)
S14A	0.0307 (2)	0.0329 (3)	0.0291 (2)	0.00466 (19)	0.00458 (19)	0.0105 (2)
O15A	0.0319 (8)	0.0483 (9)	0.0401 (8)	0.0122 (6)	0.0057 (6)	0.0173 (7)
O16A	0.0421 (8)	0.0343 (8)	0.0381 (8)	−0.0036 (6)	0.0023 (6)	0.0100 (6)
C17A	0.0311 (10)	0.0321 (10)	0.0228 (9)	0.0059 (8)	0.0039 (7)	0.0090 (8)
C18A	0.0383 (11)	0.0318 (10)	0.0365 (10)	0.0119 (9)	0.0102 (9)	0.0135 (9)
C19A	0.0407 (12)	0.0368 (11)	0.0424 (12)	0.0074 (9)	0.0095 (9)	0.0187 (10)
C20A	0.0331 (11)	0.0480 (12)	0.0322 (10)	0.0102 (9)	0.0069 (8)	0.0168 (9)
C21A	0.0408 (12)	0.0398 (11)	0.0341 (11)	0.0156 (9)	0.0110 (9)	0.0106 (9)
C22A	0.0408 (12)	0.0312 (10)	0.0313 (10)	0.0096 (9)	0.0061 (8)	0.0089 (8)
C23A	0.0424 (13)	0.0695 (17)	0.0623 (16)	0.0136 (12)	0.0242 (12)	0.0302 (14)
C24A	0.0525 (15)	0.0428 (13)	0.0467 (13)	−0.0003 (11)	−0.0080 (11)	0.0147 (11)
N25A	0.0459 (11)	0.0450 (11)	0.0450 (10)	0.0082 (9)	0.0016 (8)	0.0214 (9)
C26A	0.0372 (12)	0.0492 (13)	0.0427 (12)	0.0084 (10)	0.0009 (9)	0.0251 (11)
C27A	0.0469 (14)	0.0597 (15)	0.0655 (16)	0.0114 (12)	0.0204 (12)	0.0356 (13)
C28A	0.0531 (16)	0.0682 (18)	0.086 (2)	0.0261 (14)	0.0291 (15)	0.0396 (16)
C29A	0.0554 (16)	0.0615 (17)	0.089 (2)	0.0224 (13)	0.0143 (15)	0.0472 (16)
C30A	0.0547 (16)	0.0767 (19)	0.086 (2)	0.0191 (14)	0.0229 (14)	0.0592 (17)
C31A	0.0455 (14)	0.0672 (16)	0.0660 (16)	0.0212 (12)	0.0207 (12)	0.0429 (14)
N1B	0.0422 (10)	0.0303 (9)	0.0438 (10)	0.0015 (8)	0.0102 (8)	0.0136 (8)
C2B	0.0395 (12)	0.0339 (11)	0.0444 (12)	−0.0065 (9)	0.0114 (10)	0.0124 (10)
C3B	0.0512 (15)	0.0461 (14)	0.0567 (15)	−0.0069 (11)	0.0012 (12)	0.0177 (12)
C4B	0.0558 (16)	0.0594 (17)	0.0520 (15)	−0.0189 (13)	−0.0041 (12)	0.0124 (13)
C5B	0.0622 (17)	0.0437 (14)	0.0534 (15)	−0.0141 (13)	0.0096 (13)	0.0005 (12)
C6B	0.0520 (14)	0.0340 (12)	0.0505 (13)	−0.0044 (10)	0.0133 (11)	0.0049 (10)
C7B	0.0401 (12)	0.0301 (10)	0.0424 (12)	−0.0053 (9)	0.0153 (9)	0.0104 (9)
C8B	0.0436 (12)	0.0247 (9)	0.0379 (11)	0.0015 (8)	0.0195 (9)	0.0108 (8)
C9B	0.0546 (14)	0.0248 (10)	0.0422 (12)	0.0061 (9)	0.0222 (10)	0.0122 (9)
C10B	0.0476 (12)	0.0274 (10)	0.0437 (12)	0.0093 (9)	0.0221 (10)	0.0140 (9)
S11B	0.0623 (4)	0.0280 (3)	0.0368 (3)	0.0111 (2)	0.0123 (2)	0.0115 (2)
C12B	0.0506 (13)	0.0256 (10)	0.0406 (11)	0.0083 (9)	0.0168 (10)	0.0122 (9)
C13B	0.0392 (11)	0.0281 (10)	0.0411 (11)	0.0043 (8)	0.0174 (9)	0.0156 (9)
S14B	0.0340 (3)	0.0348 (3)	0.0507 (3)	0.0117 (2)	0.0148 (2)	0.0181 (2)
O15B	0.0367 (9)	0.0555 (10)	0.0721 (11)	0.0136 (8)	0.0064 (8)	0.0282 (9)
O16B	0.0536 (10)	0.0425 (9)	0.0589 (10)	0.0220 (7)	0.0306 (8)	0.0185 (8)
C17B	0.0343 (11)	0.0299 (10)	0.0447 (11)	0.0117 (8)	0.0120 (9)	0.0215 (9)
C18B	0.0610 (15)	0.0266 (10)	0.0550 (14)	0.0134 (10)	0.0270 (12)	0.0139 (10)
C19B	0.0692 (17)	0.0440 (13)	0.0761 (17)	0.0310 (13)	0.0469 (15)	0.0357 (13)
C20B	0.0367 (12)	0.0649 (16)	0.0832 (19)	0.0195 (12)	0.0173 (12)	0.0560 (15)
C21B	0.0460 (14)	0.0707 (17)	0.0525 (14)	−0.0068 (12)	−0.0044 (11)	0.0400 (14)
C22B	0.0463 (13)	0.0513 (13)	0.0380 (11)	0.0013 (11)	0.0035 (10)	0.0238 (11)
C23B	0.0449 (16)	0.115 (3)	0.121 (3)	0.0202 (16)	0.0284 (17)	0.089 (2)
C24B	0.0826 (19)	0.0315 (12)	0.0513 (14)	0.0178 (12)	0.0190 (13)	0.0107 (11)
N25B	0.0601 (13)	0.0320 (9)	0.0472 (11)	0.0127 (9)	0.0159 (9)	0.0185 (8)
C26B	0.0529 (14)	0.0317 (11)	0.0461 (12)	0.0184 (10)	0.0176 (10)	0.0164 (10)
C27B	0.0570 (16)	0.0425 (13)	0.0634 (16)	0.0094 (11)	0.0122 (13)	0.0268 (12)
C28B	0.0602 (17)	0.0422 (14)	0.0719 (18)	0.0189 (12)	0.0011 (14)	0.0149 (13)
C29B	0.080 (2)	0.0638 (17)	0.0458 (14)	0.0364 (16)	0.0129 (14)	0.0153 (13)
C30B	0.091 (2)	0.074 (2)	0.0540 (16)	0.0278 (18)	0.0371 (16)	0.0303 (15)
C31B	0.0611 (16)	0.0536 (15)	0.0526 (14)	0.0145 (12)	0.0261 (12)	0.0196 (12)

### (3Z)-4-Methyl-9-(4-methylbenzenesulfonyl)-N-phenyl-3H,9H-thiopyrano[3,4-b]indol-3-imine Geometric parameters (Å, º)

**Table:**

N1A—C2A	1.429 (2)	N1B—C2B	1.428 (3)
N1A—C13A	1.436 (2)	N1B—C13B	1.440 (3)
N1A—S14A	1.6659 (16)	N1B—S14B	1.6674 (18)
C2A—C3A	1.385 (3)	C2B—C3B	1.383 (3)
C2A—C7A	1.402 (3)	C2B—C7B	1.399 (3)
C3A—C4A	1.385 (3)	C3B—C4B	1.391 (4)
C3A—H3A	0.9500	C3B—H3B	0.9500
C4A—C5A	1.380 (3)	C4B—C5B	1.369 (4)
C4A—H4A	0.9500	C4B—H4B	0.9500
C5A—C6A	1.383 (3)	C5B—C6B	1.381 (4)
C5A—H5A	0.9500	C5B—H5B	0.9500
C6A—C7A	1.396 (3)	C6B—C7B	1.403 (3)
C6A—H6A	0.9500	C6B—H6B	0.9500
C7A—C8A	1.468 (3)	C7B—C8B	1.460 (3)
C8A—C9A	1.364 (3)	C8B—C9B	1.364 (3)
C8A—C13A	1.455 (3)	C8B—C13B	1.458 (3)
C9A—C10A	1.465 (3)	C9B—C10B	1.461 (3)
C9A—C24A	1.503 (3)	C9B—C24B	1.512 (3)
C10A—N25A	1.283 (3)	C10B—N25B	1.280 (3)
C10A—S11A	1.769 (2)	C10B—S11B	1.777 (2)
S11A—C12A	1.721 (2)	S11B—C12B	1.725 (2)
C12A—C13A	1.339 (3)	C12B—C13B	1.337 (3)
C12A—H12A	0.9500	C12B—H12B	0.9500
S14A—O15A	1.4255 (15)	S14B—O15B	1.4241 (17)
S14A—O16A	1.4271 (15)	S14B—O16B	1.4289 (16)
S14A—C17A	1.751 (2)	S14B—C17B	1.752 (2)
C17A—C18A	1.386 (3)	C17B—C22B	1.379 (3)
C17A—C22A	1.389 (3)	C17B—C18B	1.389 (3)
C18A—C19A	1.380 (3)	C18B—C19B	1.380 (3)
C18A—H18A	0.9500	C18B—H18B	0.9500
C19A—C20A	1.391 (3)	C19B—C20B	1.384 (4)
C19A—H19A	0.9500	C19B—H19B	0.9500
C20A—C21A	1.387 (3)	C20B—C21B	1.379 (4)
C20A—C23A	1.502 (3)	C20B—C23B	1.512 (3)
C21A—C22A	1.380 (3)	C21B—C22B	1.383 (3)
C21A—H21A	0.9500	C21B—H21B	0.9500
C22A—H22A	0.9500	C22B—H22B	0.9500
C23A—H23A	0.9800	C23B—H23D	0.9800
C23A—H23B	0.9800	C23B—H23E	0.9800
C23A—H23C	0.9800	C23B—H23F	0.9800
C24A—H24A	0.9800	C24B—H24D	0.9800
C24A—H24B	0.9800	C24B—H24E	0.9800
C24A—H24C	0.9800	C24B—H24F	0.9800
N25A—C26A	1.415 (3)	N25B—C26B	1.406 (3)
C26A—C27A	1.379 (3)	C26B—C27B	1.378 (3)
C26A—C31A	1.385 (3)	C26B—C31B	1.389 (3)
C27A—C28A	1.383 (4)	C27B—C28B	1.380 (4)
C27A—H27A	0.9500	C27B—H27B	0.9500
C28A—C29A	1.371 (4)	C28B—C29B	1.364 (4)
C28A—H28A	0.9500	C28B—H28B	0.9500
C29A—C30A	1.373 (4)	C29B—C30B	1.372 (4)
C29A—H29A	0.9500	C29B—H29B	0.9500
C30A—C31A	1.384 (4)	C30B—C31B	1.378 (4)
C30A—H30A	0.9500	C30B—H30B	0.9500
C31A—H31A	0.9500	C31B—H31B	0.9500
			
C2A—N1A—C13A	107.99 (15)	C2B—N1B—C13B	107.83 (17)
C2A—N1A—S14A	124.64 (13)	C2B—N1B—S14B	124.10 (15)
C13A—N1A—S14A	122.07 (13)	C13B—N1B—S14B	121.76 (14)
C3A—C2A—C7A	122.10 (18)	C3B—C2B—C7B	122.3 (2)
C3A—C2A—N1A	128.44 (18)	C3B—C2B—N1B	128.3 (2)
C7A—C2A—N1A	109.38 (17)	C7B—C2B—N1B	109.39 (19)
C4A—C3A—C2A	117.4 (2)	C2B—C3B—C4B	117.4 (3)
C4A—C3A—H3A	121.3	C2B—C3B—H3B	121.3
C2A—C3A—H3A	121.3	C4B—C3B—H3B	121.3
C5A—C4A—C3A	121.9 (2)	C5B—C4B—C3B	121.6 (3)
C5A—C4A—H4A	119.1	C5B—C4B—H4B	119.2
C3A—C4A—H4A	119.1	C3B—C4B—H4B	119.2
C4A—C5A—C6A	120.4 (2)	C4B—C5B—C6B	120.9 (2)
C4A—C5A—H5A	119.8	C4B—C5B—H5B	119.6
C6A—C5A—H5A	119.8	C6B—C5B—H5B	119.6
C5A—C6A—C7A	119.5 (2)	C5B—C6B—C7B	119.3 (2)
C5A—C6A—H6A	120.2	C5B—C6B—H6B	120.3
C7A—C6A—H6A	120.2	C7B—C6B—H6B	120.3
C6A—C7A—C2A	118.70 (19)	C2B—C7B—C6B	118.5 (2)
C6A—C7A—C8A	132.95 (19)	C2B—C7B—C8B	108.67 (18)
C2A—C7A—C8A	108.25 (16)	C6B—C7B—C8B	132.8 (2)
C9A—C8A—C13A	123.94 (18)	C9B—C8B—C13B	123.72 (19)
C9A—C8A—C7A	129.66 (18)	C9B—C8B—C7B	129.98 (18)
C13A—C8A—C7A	106.37 (16)	C13B—C8B—C7B	106.28 (18)
C8A—C9A—C10A	121.81 (18)	C8B—C9B—C10B	122.24 (18)
C8A—C9A—C24A	123.15 (19)	C8B—C9B—C24B	122.7 (2)
C10A—C9A—C24A	114.91 (18)	C10B—C9B—C24B	114.99 (19)
N25A—C10A—C9A	119.54 (19)	N25B—C10B—C9B	119.01 (18)
N25A—C10A—S11A	120.38 (17)	N25B—C10B—S11B	121.17 (17)
C9A—C10A—S11A	120.08 (15)	C9B—C10B—S11B	119.82 (15)
C12A—S11A—C10A	104.78 (10)	C12B—S11B—C10B	104.57 (10)
C13A—C12A—S11A	122.37 (16)	C13B—C12B—S11B	122.56 (16)
C13A—C12A—H12A	118.8	C13B—C12B—H12B	118.7
S11A—C12A—H12A	118.8	S11B—C12B—H12B	118.7
C12A—C13A—N1A	126.85 (18)	C12B—C13B—N1B	127.04 (18)
C12A—C13A—C8A	125.28 (18)	C12B—C13B—C8B	125.2 (2)
N1A—C13A—C8A	107.88 (16)	N1B—C13B—C8B	107.74 (17)
O15A—S14A—O16A	119.65 (9)	O15B—S14B—O16B	119.76 (10)
O15A—S14A—N1A	106.32 (8)	O15B—S14B—N1B	106.28 (10)
O16A—S14A—N1A	106.06 (8)	O16B—S14B—N1B	106.09 (9)
O15A—S14A—C17A	109.16 (9)	O15B—S14B—C17B	109.44 (10)
O16A—S14A—C17A	109.23 (9)	O16B—S14B—C17B	108.75 (10)
N1A—S14A—C17A	105.46 (9)	N1B—S14B—C17B	105.57 (9)
C18A—C17A—C22A	121.26 (19)	C22B—C17B—C18B	120.9 (2)
C18A—C17A—S14A	118.97 (15)	C22B—C17B—S14B	119.69 (17)
C22A—C17A—S14A	119.77 (16)	C18B—C17B—S14B	119.41 (17)
C19A—C18A—C17A	118.71 (18)	C19B—C18B—C17B	118.5 (2)
C19A—C18A—H18A	120.6	C19B—C18B—H18B	120.7
C17A—C18A—H18A	120.6	C17B—C18B—H18B	120.7
C18A—C19A—C20A	121.3 (2)	C18B—C19B—C20B	121.7 (2)
C18A—C19A—H19A	119.4	C18B—C19B—H19B	119.2
C20A—C19A—H19A	119.4	C20B—C19B—H19B	119.2
C21A—C20A—C19A	118.8 (2)	C21B—C20B—C19B	118.5 (2)
C21A—C20A—C23A	120.7 (2)	C21B—C20B—C23B	120.9 (3)
C19A—C20A—C23A	120.5 (2)	C19B—C20B—C23B	120.6 (3)
C22A—C21A—C20A	121.13 (19)	C20B—C21B—C22B	121.2 (2)
C22A—C21A—H21A	119.4	C20B—C21B—H21B	119.4
C20A—C21A—H21A	119.4	C22B—C21B—H21B	119.4
C21A—C22A—C17A	118.87 (19)	C17B—C22B—C21B	119.2 (2)
C21A—C22A—H22A	120.6	C17B—C22B—H22B	120.4
C17A—C22A—H22A	120.6	C21B—C22B—H22B	120.4
C20A—C23A—H23A	109.5	C20B—C23B—H23D	109.5
C20A—C23A—H23B	109.5	C20B—C23B—H23E	109.5
H23A—C23A—H23B	109.5	H23D—C23B—H23E	109.5
C20A—C23A—H23C	109.5	C20B—C23B—H23F	109.5
H23A—C23A—H23C	109.5	H23D—C23B—H23F	109.5
H23B—C23A—H23C	109.5	H23E—C23B—H23F	109.5
C9A—C24A—H24A	109.5	C9B—C24B—H24D	109.5
C9A—C24A—H24B	109.5	C9B—C24B—H24E	109.5
H24A—C24A—H24B	109.5	H24D—C24B—H24E	109.5
C9A—C24A—H24C	109.5	C9B—C24B—H24F	109.5
H24A—C24A—H24C	109.5	H24D—C24B—H24F	109.5
H24B—C24A—H24C	109.5	H24E—C24B—H24F	109.5
C10A—N25A—C26A	121.39 (19)	C10B—N25B—C26B	123.26 (18)
C27A—C26A—C31A	118.9 (2)	C27B—C26B—C31B	119.0 (2)
C27A—C26A—N25A	122.3 (2)	C27B—C26B—N25B	122.9 (2)
C31A—C26A—N25A	118.5 (2)	C31B—C26B—N25B	117.7 (2)
C26A—C27A—C28A	120.5 (2)	C26B—C27B—C28B	120.2 (3)
C26A—C27A—H27A	119.7	C26B—C27B—H27B	119.9
C28A—C27A—H27A	119.7	C28B—C27B—H27B	119.9
C29A—C28A—C27A	120.5 (3)	C29B—C28B—C27B	120.8 (3)
C29A—C28A—H28A	119.8	C29B—C28B—H28B	119.6
C27A—C28A—H28A	119.8	C27B—C28B—H28B	119.6
C28A—C29A—C30A	119.2 (3)	C28B—C29B—C30B	119.3 (3)
C28A—C29A—H29A	120.4	C28B—C29B—H29B	120.3
C30A—C29A—H29A	120.4	C30B—C29B—H29B	120.3
C29A—C30A—C31A	120.9 (3)	C29B—C30B—C31B	120.8 (3)
C29A—C30A—H30A	119.6	C29B—C30B—H30B	119.6
C31A—C30A—H30A	119.6	C31B—C30B—H30B	119.6
C30A—C31A—C26A	119.9 (2)	C30B—C31B—C26B	119.9 (3)
C30A—C31A—H31A	120.0	C30B—C31B—H31B	120.0
C26A—C31A—H31A	120.0	C26B—C31B—H31B	120.0
			
C13A—N1A—C2A—C3A	179.6 (2)	C13B—N1B—C2B—C3B	−179.8 (2)
S14A—N1A—C2A—C3A	25.1 (3)	S14B—N1B—C2B—C3B	−27.5 (3)
C13A—N1A—C2A—C7A	−3.8 (2)	C13B—N1B—C2B—C7B	2.8 (2)
S14A—N1A—C2A—C7A	−158.39 (14)	S14B—N1B—C2B—C7B	155.11 (15)
C7A—C2A—C3A—C4A	−0.5 (3)	C7B—C2B—C3B—C4B	−1.5 (4)
N1A—C2A—C3A—C4A	175.62 (19)	N1B—C2B—C3B—C4B	−178.6 (2)
C2A—C3A—C4A—C5A	−1.7 (3)	C2B—C3B—C4B—C5B	2.1 (4)
C3A—C4A—C5A—C6A	1.7 (4)	C3B—C4B—C5B—C6B	−0.3 (4)
C4A—C5A—C6A—C7A	0.4 (3)	C4B—C5B—C6B—C7B	−2.1 (4)
C5A—C6A—C7A—C2A	−2.5 (3)	C3B—C2B—C7B—C6B	−0.8 (3)
C5A—C6A—C7A—C8A	−178.3 (2)	N1B—C2B—C7B—C6B	176.74 (18)
C3A—C2A—C7A—C6A	2.6 (3)	C3B—C2B—C7B—C8B	−179.1 (2)
N1A—C2A—C7A—C6A	−174.23 (17)	N1B—C2B—C7B—C8B	−1.5 (2)
C3A—C2A—C7A—C8A	179.35 (18)	C5B—C6B—C7B—C2B	2.6 (3)
N1A—C2A—C7A—C8A	2.6 (2)	C5B—C6B—C7B—C8B	−179.6 (2)
C6A—C7A—C8A—C9A	−2.4 (4)	C2B—C7B—C8B—C9B	178.5 (2)
C2A—C7A—C8A—C9A	−178.5 (2)	C6B—C7B—C8B—C9B	0.6 (4)
C6A—C7A—C8A—C13A	175.8 (2)	C2B—C7B—C8B—C13B	−0.3 (2)
C2A—C7A—C8A—C13A	−0.3 (2)	C6B—C7B—C8B—C13B	−178.2 (2)
C13A—C8A—C9A—C10A	−5.6 (3)	C13B—C8B—C9B—C10B	6.0 (3)
C7A—C8A—C9A—C10A	172.34 (19)	C7B—C8B—C9B—C10B	−172.7 (2)
C13A—C8A—C9A—C24A	178.8 (2)	C13B—C8B—C9B—C24B	−177.7 (2)
C7A—C8A—C9A—C24A	−3.3 (3)	C7B—C8B—C9B—C24B	3.6 (4)
C8A—C9A—C10A—N25A	−164.4 (2)	C8B—C9B—C10B—N25B	164.6 (2)
C24A—C9A—C10A—N25A	11.6 (3)	C24B—C9B—C10B—N25B	−12.0 (3)
C8A—C9A—C10A—S11A	14.8 (3)	C8B—C9B—C10B—S11B	−15.6 (3)
C24A—C9A—C10A—S11A	−169.15 (17)	C24B—C9B—C10B—S11B	167.81 (17)
N25A—C10A—S11A—C12A	164.98 (18)	N25B—C10B—S11B—C12B	−165.89 (19)
C9A—C10A—S11A—C12A	−14.2 (2)	C9B—C10B—S11B—C12B	14.4 (2)
C10A—S11A—C12A—C13A	6.6 (2)	C10B—S11B—C12B—C13B	−5.8 (2)
S11A—C12A—C13A—N1A	−178.19 (16)	S11B—C12B—C13B—N1B	178.52 (16)
S11A—C12A—C13A—C8A	1.4 (3)	S11B—C12B—C13B—C8B	−2.8 (3)
C2A—N1A—C13A—C12A	−176.75 (19)	C2B—N1B—C13B—C12B	175.9 (2)
S14A—N1A—C13A—C12A	−21.4 (3)	S14B—N1B—C13B—C12B	22.9 (3)
C2A—N1A—C13A—C8A	3.6 (2)	C2B—N1B—C13B—C8B	−3.0 (2)
S14A—N1A—C13A—C8A	158.90 (13)	S14B—N1B—C13B—C8B	−156.04 (14)
C9A—C8A—C13A—C12A	−3.4 (3)	C9B—C8B—C13B—C12B	4.1 (3)
C7A—C8A—C13A—C12A	178.32 (19)	C7B—C8B—C13B—C12B	−176.9 (2)
C9A—C8A—C13A—N1A	176.31 (18)	C9B—C8B—C13B—N1B	−176.93 (19)
C7A—C8A—C13A—N1A	−2.0 (2)	C7B—C8B—C13B—N1B	2.0 (2)
C2A—N1A—S14A—O15A	−32.36 (18)	C2B—N1B—S14B—O15B	33.6 (2)
C13A—N1A—S14A—O15A	176.49 (15)	C13B—N1B—S14B—O15B	−177.74 (16)
C2A—N1A—S14A—O16A	−160.73 (15)	C2B—N1B—S14B—O16B	162.11 (17)
C13A—N1A—S14A—O16A	48.12 (17)	C13B—N1B—S14B—O16B	−49.25 (18)
C2A—N1A—S14A—C17A	83.47 (17)	C2B—N1B—S14B—C17B	−82.56 (19)
C13A—N1A—S14A—C17A	−67.68 (17)	C13B—N1B—S14B—C17B	66.08 (18)
O15A—S14A—C17A—C18A	36.65 (18)	O15B—S14B—C17B—C22B	128.49 (17)
O16A—S14A—C17A—C18A	169.17 (15)	O16B—S14B—C17B—C22B	−4.02 (19)
N1A—S14A—C17A—C18A	−77.22 (16)	N1B—S14B—C17B—C22B	−117.50 (17)
O15A—S14A—C17A—C22A	−142.83 (15)	O15B—S14B—C17B—C18B	−50.74 (19)
O16A—S14A—C17A—C22A	−10.31 (18)	O16B—S14B—C17B—C18B	176.76 (16)
N1A—S14A—C17A—C22A	103.30 (16)	N1B—S14B—C17B—C18B	63.27 (18)
C22A—C17A—C18A—C19A	−1.1 (3)	C22B—C17B—C18B—C19B	1.2 (3)
S14A—C17A—C18A—C19A	179.45 (15)	S14B—C17B—C18B—C19B	−179.61 (17)
C17A—C18A—C19A—C20A	−0.1 (3)	C17B—C18B—C19B—C20B	−0.6 (3)
C18A—C19A—C20A—C21A	1.2 (3)	C18B—C19B—C20B—C21B	−0.9 (4)
C18A—C19A—C20A—C23A	−177.1 (2)	C18B—C19B—C20B—C23B	178.6 (2)
C19A—C20A—C21A—C22A	−1.2 (3)	C19B—C20B—C21B—C22B	1.9 (4)
C23A—C20A—C21A—C22A	177.2 (2)	C23B—C20B—C21B—C22B	−177.6 (2)
C20A—C21A—C22A—C17A	0.1 (3)	C18B—C17B—C22B—C21B	−0.3 (3)
C18A—C17A—C22A—C21A	1.1 (3)	S14B—C17B—C22B—C21B	−179.47 (17)
S14A—C17A—C22A—C21A	−179.45 (15)	C20B—C21B—C22B—C17B	−1.3 (3)
C9A—C10A—N25A—C26A	176.5 (2)	C9B—C10B—N25B—C26B	−179.3 (2)
S11A—C10A—N25A—C26A	−2.7 (3)	S11B—C10B—N25B—C26B	1.0 (3)
C10A—N25A—C26A—C27A	74.8 (3)	C10B—N25B—C26B—C27B	−68.9 (3)
C10A—N25A—C26A—C31A	−110.9 (3)	C10B—N25B—C26B—C31B	118.7 (2)
C31A—C26A—C27A—C28A	0.7 (4)	C31B—C26B—C27B—C28B	−0.9 (3)
N25A—C26A—C27A—C28A	174.9 (2)	N25B—C26B—C27B—C28B	−173.2 (2)
C26A—C27A—C28A—C29A	0.2 (4)	C26B—C27B—C28B—C29B	0.2 (4)
C27A—C28A—C29A—C30A	−0.3 (5)	C27B—C28B—C29B—C30B	0.5 (4)
C28A—C29A—C30A—C31A	−0.6 (5)	C28B—C29B—C30B—C31B	−0.4 (4)
C29A—C30A—C31A—C26A	1.5 (4)	C29B—C30B—C31B—C26B	−0.4 (4)
C27A—C26A—C31A—C30A	−1.5 (4)	C27B—C26B—C31B—C30B	1.0 (4)
N25A—C26A—C31A—C30A	−176.0 (2)	N25B—C26B—C31B—C30B	173.7 (2)

### (3Z)-4-Methyl-9-(4-methylbenzenesulfonyl)-N-phenyl-3H,9H-thiopyrano[3,4-b]indol-3-imine Hydrogen-bond geometry (Å, º)

**Table:**

*D*—H···*A*	*D*—H	H···*A*	*D*···*A*	*D*—H···*A*
C3*A*—H3*A*···O15*A*	0.95	2.25	2.878 (3)	123
C3*B*—H3*B*···O15*B*	0.95	2.25	2.871 (3)	122
C12*A*—H12*A*···O16*A*	0.95	2.23	2.909 (2)	127
C12*B*—H12*B*···O16*B*	0.95	2.24	2.922 (3)	128
C21*A*—H21*A*···O16*B*	0.95	2.45	3.366 (3)	161
C22*A*—H22*A*···O16*A*	0.95	2.55	2.923 (2)	104
C22*B*—H22*B*···O16*B*	0.95	2.51	2.900 (3)	104
C24*A*—H24*B*···N25*A*	0.98	2.31	2.754 (4)	106
C24*B*—H24*E*···N25*B*	0.98	2.27	2.744 (3)	108
